# Cerebrospinal fluid proteomic signatures reveal *APOE* genotype-dependent lipid and immune profiles in cognitively unimpaired elderly

**DOI:** 10.21203/rs.3.rs-8605807/v1

**Published:** 2026-02-05

**Authors:** Zhiyuan Ning, Jeff Y. L. Lam, Zonghua Li, Yuka A. Martens, Sydney V. Doss, Senne B. Lageman, Maria Vassilaki, Ronald C. Petersen, Chia-Chen Liu, Michael G. Heckman, Betty M. Tijms, Takahisa Kanekiyo, Guojun Bu

**Affiliations:** 1 Division of Life Science and State Key Laboratory of Nervous System Disorders, The Hong Kong University of Science and Technology, Clear Water Bay, Hong Kong, China; 2 Department of Neuroscience, Mayo Clinic, Jacksonville, FL, United States; 3 Alzheimer Center Amsterdam, Neurology, Vrije Universiteit Amsterdam, Amsterdam UMC location VUmc, 1081HZ Amsterdam, North-Holland, The Netherlands; 4 Amsterdam Neuroscience, Neurodegeneration, 1081HZ Amsterdam, North-Holland, The Netherlands; 5 Department of Quantitative Health Sciences, Mayo Clinic, Jacksonville, FL, United States; 6 Departments of Neurology, Mayo Clinic, Rochester, MN, United States

**Keywords:** APOE, CSF proteomics, Alzheimer’s disease, lipid metabolism, neuroinflammation

## Abstract

Cerebrospinal fluid (CSF) proteomics offers insights into molecular changes in aging and Alzheimer’s disease (AD). Key AD biomarkers, in particular amyloid-β (Aβ) and tau, in CSF are strongly associated with *APOE* genotype, the strongest genetic risk determinant of AD. To investigate how *APOE* genotype influences CSF proteome across AD pathology and age, we analyzed 362 neurology-related proteins and established AD biomarkers in CSF from 145 cognitively unimpaired participants in the Mayo Clinic Study of Aging. Importantly, our cohort is uniquely balanced across *APOE* genotypes, with similar representation of *APOE2* carriers, *APOE3/3* genotype, and *APOE4* carriers. We identified several proteins, including lipid metabolism-related Lp-PLA2 and immune-related ITGAM, with strong *APOE* genotype-specific association. Notably, meta-analysis confirmed that ITGAM levels were consistently higher in *APOE4* compared to *APOE2* carriers across multiple cohorts and proteomic platforms. In addition, with increasing amyloid deposition, *APOE4* carriers exhibited stronger immune responses, reflected by elevated ITGAM, TNF-α receptors, and IL-6, whereas *APOE2* carriers showed attenuated responses. We further observed sex-specific effects among *APOE2* carriers, characterized by distinct patterns in amyloid and CXCL11 levels. These findings suggest distinct mechanisms underlying *APOE2*’s protective and *APOE4*’s detrimental effects in brain aging and AD, paving for personalized diagnostics and interventions.

## Introduction

Alzheimer’s disease (AD) is the most common cause of dementia, currently affecting more than 30 million people worldwide (1). Extensive genome-wide association studies (GWAS) and meta-analyses (2) have identified the apolipoprotein E (*APOE*) ε4 gene allele (*APOE4*) as the strongest genetic risk factor for AD, with *APOE4* present in approximately 40–65% of AD patients (3). In contrast, the *APOE2* allele is associated with a reduced risk of developing AD compared to the reference *APOE3* allele, and is linked to increased longevity (4, 5). ApoE plays pivotal roles in lipid metabolism, amyloid-β (Aβ) clearance and aggregation, immune responses, vascular pathology, and tau-mediated neurodegeneration (6–11), highlighting its critical contribution to AD pathogenesis and aging.

AD is clinically characterized by the progressive decline of memory and cognitive function. In addition to clinical characteristics, recent advances in AD diagnosis and staging incorporate measurements of fluid biomarkers and neuroimaging modalities to enhance diagnostic precision. This integrated framework, often referred to as the ATN system, evaluate Aβ deposition (A), tau pathology (T), and neurodegeneration (N) (12). While positron emission tomography (PET) imaging provides spatial and topographical information, cerebrospinal fluid (CSF) biomarker analysis, encompassing simultaneous measurements of proteins including Aβ42, total tau (T-tau), phosphorylated tau181 (P-tau181), tau217 (P-tau217), and tau 231 (P-tau 231), and neurofilament light chain (NfL), demonstrates comparable or slightly superior sensitivity and accuracy in detecting early stages of AD pathology (13, 14).

Despite significant advances in AD diagnostics, there remains an urgent need to identify effective biomarkers that capture the multifactorial changes in the brain before the onset of clinical symptoms. Such markers are essential for enabling early diagnosis or even prediction of cognitive decline during the presymptomatic stage, which is important for timely preventive or therapeutic interventions. Our previous study and recent research have focused on identifying novel CSF biomarkers associated with synaptic plasticity, blood-brain barrier integrity, and immune system activation (15–17) reflecting additional key pathological processes that may or may not depend on amyloid and tau pathology. While these emerging markers hold promise for improving early detection and understanding of AD, their robustness is still limited by individual variability driven in part by genetic factors and biological differences such as *APOE* genotype, age, and sex.

One critical gap in studies of CSF proteomes is the limited representation of *APOE2* carriers due to its lower allele frequency and association with reduced AD risk. To investigate the influence of *APOE* genotype and sex on CSF biomarkers in response to AD pathogenesis and aging, we analyzed 362 neurological biomarkers in CSF collected from 145 cognitively unimpaired, *APOE* genotype-balanced participants in the Mayo Clinic Study of Aging (MCSA) cohort using proximity extension assay technology (15). Established AD biomarkers, including Aβ42, T-tau, P-tau181, and NfL, were also measured. We further validated our key findings in additional cohorts, strengthening the robustness of our study outcomes. This study aims to explore the relationships between neurological biomarkers, *APOE* genotype, and sex, as well as to evaluate potential interactions between *APOE* genotype and sex in relation to clinical characteristics and their association with neurological biomarkers.

## Results

### Effect of *APOE* genotype on AD biomarkers and neurological proteins in the CSF

**Figure 1.**
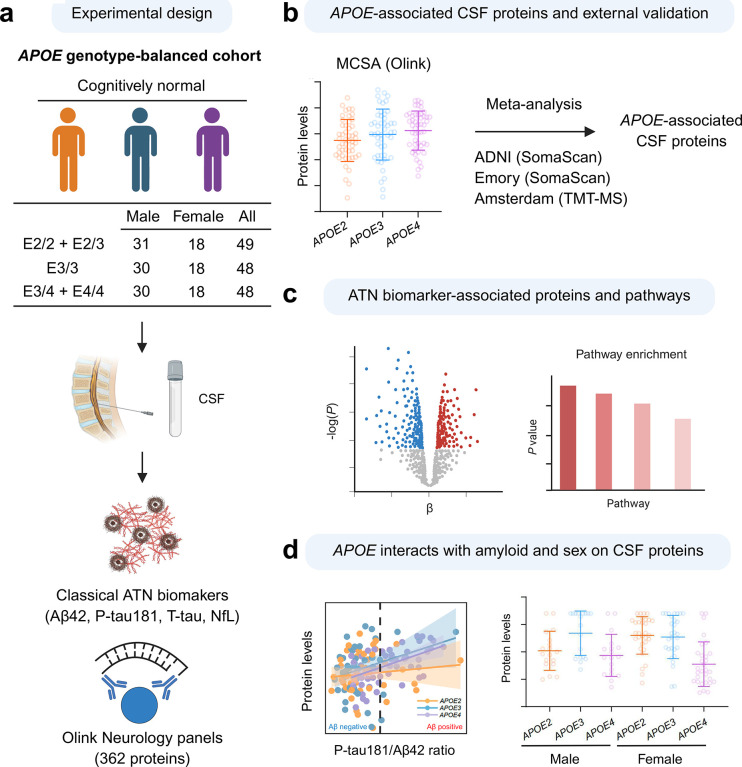
Schematic overview of the study. (a) CSF samples from an *APOE* genotype–balanced cohort were analyzed for ATN biomarkers (Aβ42, P-Tau181, T-tau, and NfL) and profiled using the Olink Neurology panel (362 neurological proteins). (b) Multiple linear regression identified *APOE*-associated proteins, which were subsequently validated in independent cohorts and combined through meta-analysis. (c) ATN-associated proteins were identified and underwent pathway enrichment analysis, revealing molecular changes related to AD pathology. (d) Interactions between *APOE* genotype and amyloid deposition (P-tau181/Aβ42 ratio) or sex on CSF neurological protein levels were further examined.

**Figure 2.**
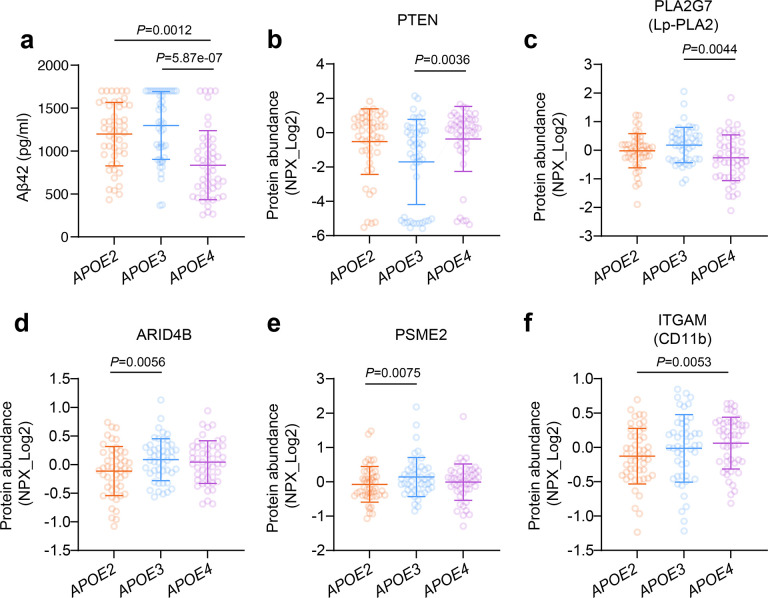
The effect of *APOE* genotype on AD biomarkers and CSF neurological proteins. Scatter plots showing the levels of Aβ42 (a), PTEN (b), PLA2G7 (c), ARID4B (d), PSME2 (e), ITGAM (f) by *APOE* genotype (mean ± SD). All analyses used linear regression models adjusted for age, sex, years of education, BMI, smoking status, hypertension, diabetes, and dyslipidemia.

A total of 145 unrelated, non-Hispanic/Latino Caucasian subjects from the MCSA who had CSF available and were cognitively unimpaired were included in this study (Figure 1). We collected comprehensive demographic and clinical data, including *APOE* genotype, age, sex, years of education, body mass index (BMI), smoking status, hypertension, diabetes, and dyslipidemia. The *APOE* genotype distribution was as follows: 2 subjects with ε2/ε2, 47 subjects with ε2/ε3, 48 subjects with ε3/ε3, 44 subjects with ε3/ε4, and 4 subjects with ε4/ε4. Due to the limited number of ε2/ε2 and ε4/ε4 individuals, we categorized *APOE* genotypes as *APOE2* (ε2/ε2 or ε2/ε3, N=49), *APOE3* (ε3/ε3, N=48), and *APOE4* (ε3/ε4 or ε4/ε4, N=48) in all analyses. Importantly, our cohort features a notably large representation of *APOE2* carriers alongside *APOE3* and *APOE4* carriers, representing a distinctive strength of this study that provides a valuable opportunity to investigate this less common protective genotype in AD. Demographic characteristics, including age, sex, education, were comparable across groups. Nevertheless, *APOE2* carriers had a higher BMI but lower prevalence of dyslipidemia (Table 1). Regarding AD biomarkers, *APOE4* carriers showed significantly reduced Aβ42 levels (Figure 2a), indicating greater Aβ deposition even in the absence of cognitive impairment, while the levels of P-tau181, T-tau, and NfL were comparable across groups (Table S1).

To evaluate differences in CSF protein levels across *APOE* groups, we utilized the Olink proximity extension assay neurology panel, which includes 362 proteins associated with neurological diseases and neurobiological processes. Using linear regression models adjusted for all the demographic and clinical variables, there were no significant associations between *APOE* group and proteins after applying a Bonferroni correction for multiple testing. However, five proteins demonstrated suggestive significance (*P* < 0.01) (Figure 2b–f, Table S2). Compared with *APOE3* individuals, *APOE4* carriers showed elevated levels of phosphatase and tensin homolog (PTEN; β = 1.40, 95% CI: 0.47–2.32, *P* = 0.0036) and reduced levels of lipoprotein-associated phospholipase A2 (Lp-PLA2; also known as PLA2G7; β = −0.45, 95% CI: −0.75 to −0.14, *P* = 0.0044) (Figure 2b–c, Table S2). *APOE2* carriers, relative to *APOE3*, exhibited lower levels of AT-rich interaction domain 4B (ARID4B; β = −0.26, 95% CI: −0.44 to −0.079, *P* = 0.0056) and proteasome activator subunit 2 (PSME2; β = −0.33, 95% CI: −0.58 to −0.092, *P* = 0.0075) (Figure 2d–e, Table S2). In addition, *APOE4* carriers, compared with *APOE2*, had higher levels of integrin subunit alpha M (ITGAM, also known as CD11b; β = 0.25, 95% CI: 0.077–0.43, *P* = 0.0053) (Figure 2f, Table S2).

To confirm the significance of our findings, we examined the levels of these five CSF proteins in three additional cohorts: the Alzheimer’s Disease Neuroimaging Initiative (ADNI) (18, 19), the Emory cohort (20), and the Alzheimer Center Amsterdam (Amsterdam) cohort (16, 21). CSF protein levels of cognitively unimpaired individuals in these cohorts were profiled using orthogonal platforms, including SomaLogic proteomics (19, 20) and untargeted TMT-based mass spectrometry (16). We then performed regression analyses for the five proteins across the available cohorts, followed by a meta-analysis. ITGAM levels were significantly higher in *APOE4* carriers compared with *APOE2* carriers in both the Somascan-based ADNI cohort (β= 0.11, 95% CI: 0.0089–0.21, *P*= 0.033) and the TMT-based Amsterdam cohort (β= 0.60, 95% CI: 0.085–1.1, *P*= 0.023) (Table S12). The meta-analysis confirmed a significant association under both fixed- and random-effects models (SMD = 0.52, 95% CI: 0.25–0.80, *P* < 0.001), with no heterogeneity (τ^2^ = 0, I^2^ = 0) (Supplementary Figure 1, Table S12). For PLA2G7, the fixed-effects model indicated a significant association (SMD = −0.23, 95% CI: −0.40 to −0.064, *P* = 0.007); however, the association showed reduced significance under the random-effects model (SMD = −0.23, 95% CI: −0.48–0.010, *P* = 0.060), reflecting moderate heterogeneity across studies (τ^2^ = 0.029, I^2^ = 47%) (Supplementary Figure 1, Table S13).

### Identification of CSF neurological proteins and pathways associated with ATN biomarkers

We first examined the relationships among CSF ATN biomarkers in our cohort. CSF Aβ42 showed no significant association with P-tau181 (*P* = 0.11) (Supplementary Figure 2a, Table S14) but a positive association with T-tau (*P* = 0.0019) (Supplementary Figure 2b, Table S14). These findings suggest that, in this cognitively unimpaired and community-based cohort, reduced Aβ42 levels have not yet resulted in a marked increase in tau, reflecting an early amyloid-driven stage of AD pathology. Both P-tau181 and T-tau showed stronger associations with NfL than Aβ42, indicating a closer link between tau pathology and neurodegeneration (Supplementary Figure 2c–e, Table S14). NfL levels further modulated the association between Aβ42 and tau biomarkers (Supplementary Figure 2f–g, Table S14). In individuals with low NfL, Aβ42 was positively associated with P-tau181 and T-tau, supporting the view that early Aβ accumulation does not yet lead to an increase in tau.

**Figure 3.**
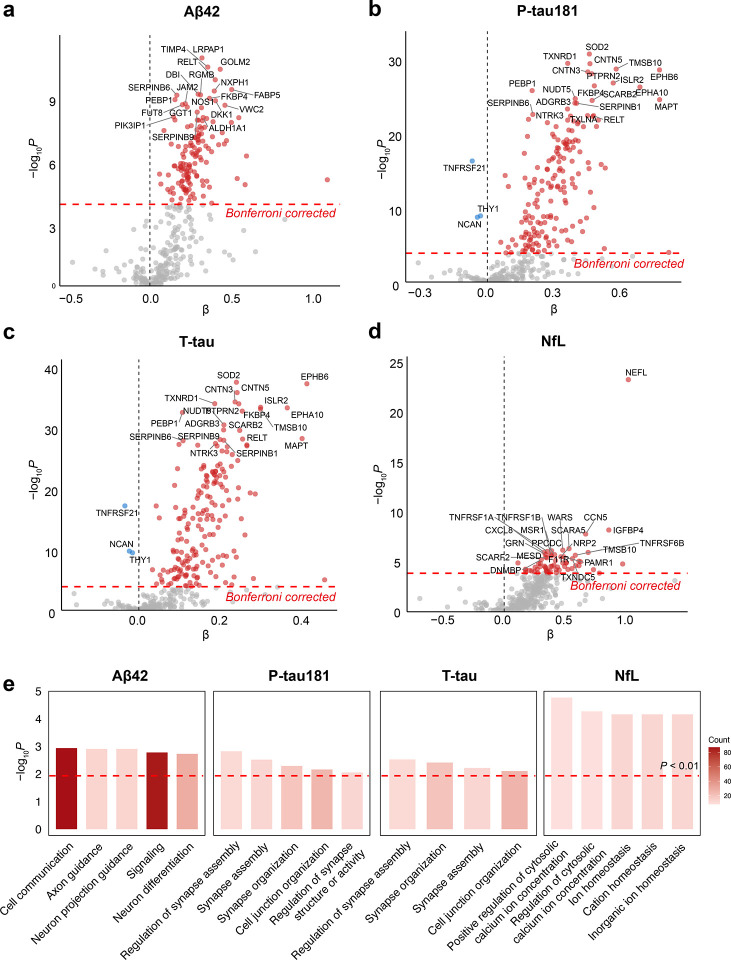
Identification of neurological proteins and pathways associated with ATN biomarkers. (a–d) Volcano plots showing the association of CSF proteins with ATN biomarkers, Aβ42 (a), P-tau181 (b), T-tau (c), and NfL (d), using linear regression models adjusted for *APOE* genotype, age, sex, years of education, BMI, smoking status, hypertension, diabetes, and dyslipidemia. β values represent the change in the mean protein level corresponding to a 500-unit increase in Aβ42, a 10-unit increase in P-tau181, a 50-unit increase in T-tau, and a doubling of NfL. The top 20 significant proteins are labeled. (e) Bar plot of GO BP pathways enriched with neurological proteins significantly associated with Aβ42, P-tau181, T-tau, and NfL. Bar color represents the number of proteins enriched in each pathway, and the bar height indicates the −log10(*P* value).

We next investigated the associations between 362 neurological proteins in CSF and established AD biomarkers through an extensive association analysis. After Bonferroni correction, a substantial number of proteins demonstrated significant associations: 139 with Aβ42 (Figure 3a, Table S3), 176 with P-tau181 (Figure 3b, Table S4), 187 with T-tau (Figure 3c, Table S5), and 63 with NfL (Figure 3d, Table S6).

We conducted Gene Ontology biological process (GO BP) pathway analysis for proteins significantly associated with ATN biomarkers (Figure 3e, Table S7). Our analysis identified several key pathways related to Aβ42, P-tau181, and T-tau, involved in axon guidance and synaptic processes. These pathways included proteins such as APP, SEMA4D, and the cell adhesion molecules contactins CNTN4 and CNTN5. Additionally, EFNA4, EPHA10, and EPHB6 (Eph/ephrin signaling proteins) were enriched for their roles in axonal guidance, synapse formation, and neuronal plasticity. NfL-associated proteins were enriched in pathways related to ion regulation, with proteins like CALCA, STC2, and NOS1 involved in regulating calcium ion homeostasis. In addition, proteins linked to Aβ42 and NfL were particularly enriched in immune cell chemotaxis and migration pathways. Key proteins such as CXCL8, CXCL13, and CCL19 promote the chemotaxis and migration of neutrophils and lymphocytes (22–24). CX3CL1 regulates myeloid and T-cell migration (25), while CD99 facilitates immune-cell infiltration and adhesion (26). These findings highlight the central role of immune responses in the pathophysiology of AD associated with ATN biomarkers.

### Effect of *APOE* genotype on CSF neurological proteins in response to amyloid pathology

**Figure 4.**
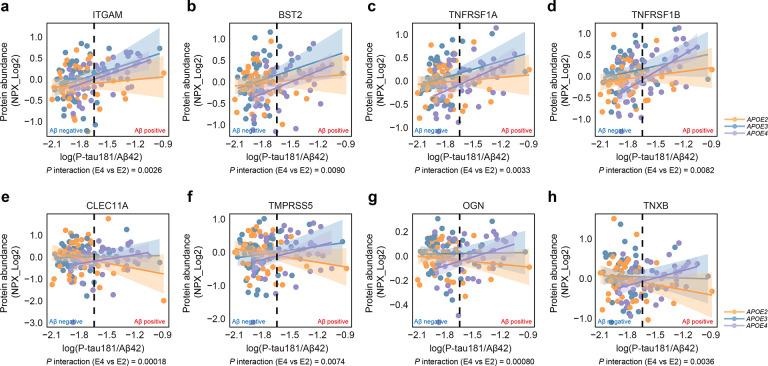
Effect of *APOE* genotype on CSF neurological protein responses to amyloid pathology. Scatter plots of ITGAM (a), BST2 (b), TNFRSF1A (c), TNFRSF1B (d), CLEC11A (e), TMPRSS5 (f), OGN (g), and TNXB (h), illustrating the relationship between the P-tau181/Aβ42 ratio and protein abundance across *APOE* genotypes. Yellow, blue, and purple points and regression lines represent *APOE2*, *APOE3*, and *APOE4* carriers, respectively. The vertical line indicates log_10_(0.023), the cutoff for amyloid positivity based on the P-tau181/Aβ42 ratio. *P* values for the interaction between amyloid positivity (P-tau181/Aβ42 ratio as a binary variable) and *APOE* genotype were derived from linear regression models adjusted for age, sex, years of education, BMI, smoking status, hypertension, diabetes, and dyslipidemia.

Given the well-established association between *APOE4* and increased cerebral amyloid deposition, we further investigated the effect of *APOE* genotype on the response of different neurological proteins to amyloid pathology. Recent study suggests that the CSF P-tau181/Aβ42 ratio reflects cerebral amyloid burden better than Aβ42 alone (27, 28). Consistent with this, we also observed a bimodal distribution of the P-tau181/Aβ42 ratio in our cohort (Supplementary Figure 3a). Therefore, we used P-tau181/Aβ42 ratio as the primary indicator for cerebral amyloid deposition. We used an Elecsys P-tau181/Aβ42 ratio cutoff of 0.023 for amyloid positivity, as previously established in the MCSA cohort (27, 28). Using this cutoff, 8 of 49 *APOE2* carriers, 6 of 47 *APOE3* individuals, and 28 of 48 *APOE4* carriers were classified as “amyloid-positive”. We next explored the interaction between *APOE* genotype and amyloid positivity on neurological protein levels. We found that *APOE4* carriers exhibited a more pronounced immune response (Figure 4a–d, Table S8), including elevated levels of ITGAM (*APOE4* vs *APOE2* β= 0.59, 95% CI: 0.21–0.98, *P* = 0.0026) and BST2 (involved in interferon pathways) (*APOE4* vs *APOE2* β= 0.61, 95% CI: 0.16–1.06, *P* = 0.0090), as well as TNF-α receptors TNFRSF1A (*APOE4* vs *APOE2* β= 0.61, 95% CI: 0.21–1.00, *P* = 0.0033) and TNFRSF1B (*APOE4* vs *APOE2* β= 0.60, 95% CI: 0.16–1.04, *P* = 0.0082), which were rapidly upregulated with increasing amyloid deposition. In contrast, *APOE2* carriers showed more modest changes. Specifically, *APOE2* carriers demonstrated downregulation of proteins involved in extracellular matrix remodeling (Figure 4e–h, Table S8), including the secreted sulfated glycoprotein CLEC11A (*APOE4* vs *APOE2* β= 1.31, 95% CI: 0.64–1.97, *P* = 0.00018), the serine protease TMPRSS5 (*APOE4* vs *APOE2* β= 0.77, 95% CI: 0.21–1.32, *P* = 0.0074), the keratan sulfate proteoglycan osteoglycin (OGN) (*APOE4* vs *APOE2* β= 0.25, 95% CI: 0.11–0.39, *P* = 0.00080), and the glycoprotein tenascin-X (TNXB) (*APOE4* vs *APOE2* β=0.67, 95% CI: 0.23–1.12, *P* = 0.0036).

We also separately compared differentially abundant proteins (*P* < 0.01) between amyloid-positive and amyloid-negative individuals stratified by *APOE* genotype (Supplementary Figure 3b–d, Table S9). *APOE2* carriers with amyloid deposition showed downregulation of the chemokines CXCL8 (β= −0.57, 95% CI: −0.91 to −0.24, *P* = 0.0014) and CXCL13 (β= −1.09, 95% CI: −1.78 to −0.40, *P* = 0.0028), while *APOE3* individuals showed upregulation of HAVCR2 (β= 0.65, 95% CI: 0.21–1.10, *P* = 0.0052), and *APOE4* carriers with amyloid deposition exhibited upregulation of the myeloid cell receptors ITGAM (β= 0.31, 95% CI: 0.092–0.54, *P* = 0.0068) and CSF2RA (β= 0.64, 95% CI: 0.16–1.12, *P* = 0.0099). These findings further demonstrate distinct immune responses to amyloid across different *APOE* genotypes.

In addition to treating the P-tau181/Aβ42 ratio as a binary variable, we also explored its interaction with *APOE* genotype as a continuous variable. Beyond the aforementioned proteins, we observed that IL-6, a potent pro-inflammatory cytokine, increased significantly with rising P-tau181/Aβ42 ratios in *APOE4* carriers compared to *APOE2* carriers (*APOE4* vs *APOE2* β= 2.03, 95% CI: 0.76–3.31, *P* = 0.0021) (Table S10). Overall, these findings suggest that *APOE2* is associated with a distinct amyloid response, differing from the response observed in *APOE3* or *APOE4* carriers.

### Sex modulates the effects of *APOE* genotype on amyloid and CSF neurological proteins

**Figure 5.**
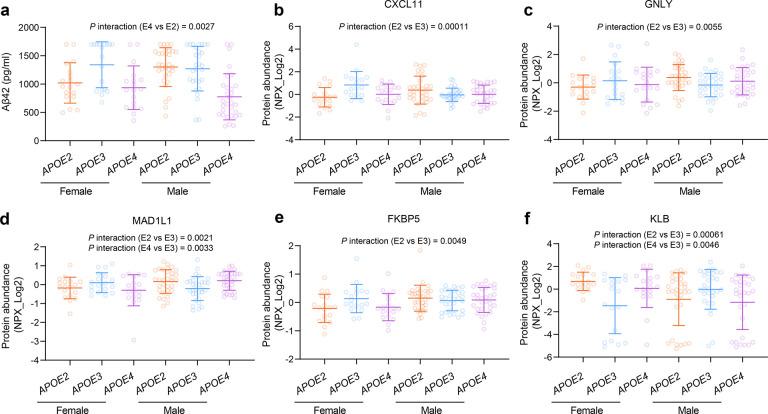
Sex modulates the effects of *APOE* genotype on amyloid and neurological proteins. Scatter plots showing the levels of CSF Aβ42 (a), CXCL11 (b), GNLY (c), MAD1L1 (d), FKBP5 (e), and KLB (f) stratified by sex and *APOE* genotype (mean ± SD). *P* values for the interaction between sex and *APOE* genotype were calculated by linear regression models adjusted for age, sex, years of education, BMI, smoking status, hypertension, diabetes, and dyslipidemia.

Since sex is an established risk factor for AD and modulates the effect of *APOE* on AD risk, we investigated the interaction between *APOE* genotype and sex on CSF AD biomarkers and neurological proteins. We found that both sex and *APOE* genotype jointly regulate CSF Aβ42 levels (*APOE4* vs *APOE2* β= −496.88, 95% CI: −816.17 to −177.68, *P* = 0.0032), with females carrying *APOE2* showing lower CSF Aβ42 levels (Figure 5a, Table S10). For neurological proteins, a total of five proteins exhibited suggestive significance (*P* values < 0.01) in the interaction between sex and *APOE* genotype. These included the immune-related molecule chemokine CXCL11 (*APOE2* vs *APOE3* β = 1.77, 95% CI: 0.91–2.64, *P* = 0.00011), granulysin (GNLY) (*APOE2* vs *APOE3* β = 1.23, 95% CI: 0.37–2.08, *P* = 0.0055). Additionally, we identified the protein cochaperone FKBP5 (*APOE2* vs *APOE3* β = 0.54, 95% CI: 0.17–0.92, *P* = 0.0049), cell cycle checkpoint protein MAD1L1 (*APOE2* vs *APOE3* β = 0.83, 95% CI: 0.31–1.35, *P* = 0.0021; *APOE4* vs *APOE3* β = 0.84, 95% CI: 0.29–1.39, *P* = 0.0033), and the metabolism-related protein KLB (*APOE2* vs *APOE3* β = −3.13, 95% CI: −4.87 to −1.38, *P* = 0.00061; *APOE4* vs *APOE3* β = −2.70, 95% CI: −4.55 to −0.86, *P* = 0.0046) as showing significant interactions between sex and *APOE* genotype (Figure 5b–f, Table S11). These findings highlight the sex-specific modulation of CSF biomarkers by *APOE* genotype, suggesting that the effects of *APOE* on amyloid metabolism, immune signaling, protein homeostasis, and metabolic pathways differ between sexes.

## Discussion

CSF proteomics has become an increasingly valuable approach for uncovering molecular alterations linked to neurodegenerative diseases (19, 29, 30). In this study, the use of a cognitively unimpaired cohort with balanced *APOE* genotypes provides a unique opportunity to detect subtle molecular changes that emerge during the presymptomatic stage of AD.

While the pathological role of *APOE4* is well established due to its high prevalence in AD, the protective mechanisms of *APOE2* remain less well understood, primarily because of its low population frequency (4, 31). To address this gap, the cohort in this study includes balanced representation across *APOE* genotypes, enabling the identification of *APOE* genotype-specific molecular signatures. This design provides valuable insights into early diagnostic and predictive biomarkers, as well as the distinct pathogenic and protective mechanisms underlying AD across *APOE* genotypes.

Since the primary function of apoE is to transport lipids and its isoforms differ in lipid-carrying capacity (32–34), aberrant lipid metabolism in CSF may represent a critical pathway through which *APOE* genotype influences AD pathophysiology. Indeed, our findings reveal a significant association between *APOE* genotype and lipoprotein-associated phospolipase A2 (Lp-PLA2, *PLA2G7*). Peripherally, Lp-PLA2 is a secreted enzyme circulating in plasma bound to LDL and HDL, where it hydrolyzes oxidized LDL into pro-inflammatory lysophosphatidylcholines (lysoPC) and oxidized non-esterified fatty acids (oxNEFAs), contributing to vascular inflammation and dysfunction, including pericyte loss and blood-brain barrier disruption (35, 36). Intriguingly, our study demonstrated significantly reduced CSF Lp-PLA2 levels in *APOE4* carriers compared to *APOE2* and *APOE3*. In *APOE4* AD patients, significant lipid deposition in the brain may lead to lower CSF Lp-PLA2 levels, as Lp-PLA2 might increasingly deposit alongside these lipids and amyloid (37). Additionally, the poor lipidation of apoE4 might also contribute to the reduced Lp-PLA2 in the CSF (38).

Recent research highlights the protective roles of inhibiting Lp-PLA2 in countering age-related inflammation, improving metabolic health, and extending lifespan (39). Furthermore, a phase II clinical trial for AD suggests that the Lp-PLA2 inhibitor Rilapladib may improve cognitive functions, positioning Lp-PLA2 as a potential therapeutic target for *APOE4* and AD (40). However, further investigation is needed to elucidate the precise role of Lp-PLA2 in the brain and its intricate relationship with *APOE4*.

In addition to its established function as a lipid transporter, recent studies increasingly highlight the role of apoE in modulating immune response (41–43). *APOE* genotype differentially regulates microglia function, a process pivotal to AD pathogenesis (44). Improving effective phagocytosis while reducing detrimental side effects, such as excessive inflammation and synaptic engulfment, remains a crucial therapeutic strategy in AD (45–47). Our study revealed that CSF levels of ITGAM, also known as CD11b, are dependent on *APOE* genotype, with the highest levels in *APOE4* carriers and the lowest in *APOE2* carriers. ITGAM is typically expressed on myeloid cells and primarily in microglia within the brain (48, 49). Large-scale CSF proteomics have previously shown that ITGAM has the strongest positive associations with global Aβ-PET levels, suggesting its levels are primarily amyloid-driven (48). Our interaction analysis further demonstrated that, even with high amyloid loads, the ITGAM levels remained lower in *APOE2* carriers. ITGAM, expressed on microglia and macrophages, plays a key role in microglial activation, phagocytosis, and cell adhesion (50, 51). In AD mouse models, ITGAM acts as a receptor for fibrinogen, inducing excessive inflammatory responses and synaptic engulfment, leading to cognitive impairment (52). The precise mechanism by which *APOE* regulates ITGAM remains unclear. Previous studies show that overexpression of the LDL receptor decreases ITGAM levels in mice and shifts microglial metabolism towards catabolism rather than anabolism (53, 54). As such, one hypothesis is that apoE isoforms differentially modulate microglial metabolism, thereby impacting ITGAM expression and/or function. Further studies, including both *in vitro* and *in vivo*, are warranted to directly evaluate how distinct apoE isoforms impact microglial ITGAM expression and related functional outcomes. Additionally, we observed that as amyloid deposition increased, CSF IL-6 levels rose most prominently in *APOE4* carriers, while *APOE2* carriers showed a downward trend. A similar trend was seen with caspase 10, a protein associated with apoptosis and inflammation. These findings suggest that the *APOE* genotype influences distinct immune responses to amyloid, with *APOE4* being associated with a stronger inflammatory response compared to *APOE2*.

Our study revealed that sex may modulate the effects of *APOE* genotype on amyloid and immune responses, aligning with sex as a significant AD risk factor interacting with *APOE* (55, 56). Previous studies show that *APOE2* selectively protects non-Hispanic White men from cognitive decline, with male exhibiting a slower rate of cognitive decline than females (57). In our study cohort with balanced *APOE* genotype representations, *APOE2* females appeared to have lower CSF Aβ42 levels than *APOE2* males. Further large-scale studies are needed to determine if this difference correlates with increased cerebral Aβ deposition and longitudinal cognitive changes.

Despite these valuable insights, our study has several limitations. This study was conducted within the MCSA, a cohort largely composed of individuals from a geographically defined and relatively homogeneous population in Olmsted County, Minnesota, which may limit the generalizability of our findings to more diverse populations. We, however, address this potential limitation by validating our results against three external cohorts with participants from more diverse geographical backgrounds. Furthermore, by focusing on a targeted panel of 362 neurological proteins, our study may not have captured full diversity of CSF proteomes. Employing a broader, unbiased proteomic approach would add additional insights into the impact of *APOE* genotype in cognitively unimpaired individuals.

In conclusion, our study utilized targeted proteomics within a community-based, *APOE* genotype-balanced cohort to provide crucial insights into the influence of *APOE* on aging and AD during the presymptomatic stage. We found that *APOE4* carriers exhibit significantly increased amyloid deposition, aberrant lipid metabolism, heightened immune activation in the absence of cognitive impairment. Conversely, *APOE2* carriers demonstrate a more regulated immune response despite elevated amyloid levels. These findings enhance our understanding of how the *APOE* genotype differentially affects AD. This new knowledge could lead to improvements in early diagnosis and predictive models, help identify novel therapeutic targets, and guide the development of personalized, *APOE*-targeted treatment strategies to prevent or delay the onset of AD.

## Methods

### Participants

This study included 145 *APOE* genotype-balanced and cognitively unimpaired participants (CDR global score = 0, >60 years old) from the Mayo Clinic Study of Aging (MCSA). The MCSA is a prospective, community-based cohort designed to investigate the prevalence, incidence, and risk factors for mild cognitive impairment (MCI) and dementia. All participants were unrelated, white individuals from Olmsted County, Minnesota, who had available CSF samples (15, 58). We included 49 *APOE2* carriers, 48 *APOE3* homozygotes, and 48 *APOE4* carriers. Additionally, we collected data on their age, sex, years of education, BMI, smoking status, hypertension, diabetes, and dyslipidemia. Detailed demographic information is presented in Table 1.

For the replication analyses, data were drawn from three cohorts: ADNI, Emory, and Amsterdam. Detailed demographic information is in Table 2. The ADNI cohort serially collected clinical, genetic, imaging, biological, and neuropsychological measures to study MCI and early AD progression (59). From 707 participants with CSF proteomic data, 164 cognitively normal elderly individuals (CDR = 0) were retained after excluding *APOE* ε2/ε4 genotypes. This included 22 *APOE2* carriers, 102 *APOE* ε3/ε3 genotype carriers, and 40 *APOE4* carriers (19, 29). The Emory cohort utilized CSF samples from the Emory Goizueta Alzheimer’s Disease Research Center (ADRC) and Emory Healthy Brain Study (EHBS) (20). After excluding the *APOE* ε2/ε4 genotype and selecting those with MoCA scores > 26, 104 cognitively normal individuals were included (20 *APOE2* carriers, 59 *APOE* ε3/ε3 genotype carriers, and 25 *APOE4* carriers). The Amsterdam cohort, from the Alzheimer Center Amsterdam, including Amsterdam Dementia Cohort (ADC) (60), EMIF-AD preclinAD (61) and 90+ studies (62), and Amsterdam site participants who co-enrolled in the ADC biobank and the EPAD study (63), enrolled 275 cognitively normal participants after excluding *APOE* ε2/ε4 participants, comprising 22 *APOE2* carriers, 147 *APOE* ε3/ε3 genotype carriers, and 106 *APOE4* carriers (16).

### CSF measurements for AD biomarkers and Olink neurological proteins

Cerebrospinal fluid (CSF) samples were collected via lumbar puncture. To remove leukocytes, samples were centrifuged at 2000g for 10 minutes, with the initial 1–2 ml discarded to avoid blood contamination. CSF Aβ42, total tau, and P-tau181 levels were measured using automated electrochemiluminescence Elecsys immunoassays (Roche Diagnostics), while CSF NfL levels were determined by enzyme-linked immunosorbent assay (ELISA). Additionally, 362 neurological proteins relevant to neurological disease and neurobiology research were measured using the Olink Neurology panels via a proximity extension assay. This assay involves protein-specific antibodies conjugated to oligonucleotide tags, where epitope-specific binding leads to the hybridization of paired oligonucleotide DNA tags. Samples are then processed through quantitative polymerase chain reaction to generate normalized protein expression (NPX) values on log2 scales.

### Statistical analysis

All statistical analyses were conducted using R statistical software (version 4.2.1). Demographics and clinical characteristics were summarized as numbers (percentages) or means (standard deviations). Given the small numbers of *APOE* ε2/ε2 and ε4/ε4 participants, *APOE* genotypes were grouped for analysis into *APOE2* (ε2/ε2 or ε2/ε3, N=49), *APOE3* (ε3/ε3, N=48), and *APOE4* (ε3/ε4 or ε4/ε4, N=48). Comparisons across *APOE* genotypes were performed using Fisher’s exact tests or Kruskal-Wallis tests.

The Olink assay provided normalized protein expression (NPX) values for 362 proteins, with no missing values, suitable for downstream analysis. We used multivariable linear regression models to explore the relationship between *APOE* genotype and established AD biomarkers, as well as CSF neurological proteins. The NfL and P-tau181/Aβ42 ratio were log-transformed due to the skewed distributions. *APOE* genotype was treated as a categorical variable, and models were adjusted for age, sex, years of education, body mass index (BMI), smoking status, hypertension, diabetes, and dyslipidemia. We also examined the interaction between *APOE* genotype and the P-tau181/Aβ42 ratio (as both a binary and continuous variable) or sex on CSF neurological proteins, adjusting for all covariates. We utilized a Bonferroni correction for multiple testing in order to account for 362 different neurological biomarkers that were assessed for association or interaction with clinical characteristics and established biomarkers, after which *P* values <0.00014 were considered as statistically significant. However, since strict correction for multiple testing, while controlling the likelihood of a type I error (i.e., false-positive finding), increases the probability of a type II error (i.e., a false-negative finding), *P* values <0.01 were considered as displaying suggestive evidence of an association. One sample was excluded from the relevant analyses due to the absence of all AD biomarker data, and an additional ten samples were excluded due to absent NfL measurements.

For the replication cohort, the quantification of five proteins (ITGAM, PLA2G7, PSME2, ARID4B, and PTEN) involved values obtained from different platforms: log-transformed relative fluorescence units (RFU) from the Somascan platform (ADNI and Emory cohorts) and scaled log-transformed quantitative protein values from TMT-MS (Amsterdam cohort). We performed multiple linear regression on the available protein data, with the ADNI cohort adjusting for age, sex, race, and years of education; the Emory cohort adjusting for age, sex, and race; and the Amsterdam cohort adjusting for age, sex, and years of education. A *P* value below 0.05 was considered significant.

We conducted the meta-analysis using the metafor package (version 4.8.0) in R. For each protein in each cohort, we calculated the standardized mean difference (SMD) based on the mean, standard deviation, and sample size in each group. We pooled the SMD estimates using both fixed-effects and random-effects models, calculating a summary SMD estimate with 95% CI, prediction interval, and heterogeneity measures (τ, Q, and I^2^). The results were visualized in a forest plot.

### Pathway enrichment analysis

Gene Ontology (GO) pathway enrichment analysis was performed using the enrichGO function from the clusterProfiler R package (version 4.4.4) (64). This analysis aimed to identify enriched pathways within the Gene Ontology, focusing on biological process (BP). The Olink neurological proteins measured in this study were used as the background gene set (input as the universe). Pathways with *P* values less than 0.01 were considered significant, and the top 5 enriched pathways are highlighted in the figure.

## Supplementary Material

1 **Supplementary figure 1: Cross-cohort and cross-platform meta-analysis.** Forest plot showing the standardized mean difference (SMD) of ITGAM (a), PLA2G7 (b), PSME2 (c), and PTEN (d) across different cohorts (MCSA, ADNI, Amsterdam, and Emory). SMD values represent the effect sizes for each protein, and heterogeneity was assessed across the cohorts. *P* values were calculated using a mixed-effects model.
**Supplementary figure 2: Associations among ATN biomarkers** (a-e). Scatter plots show the relationships between Aβ42 and P−tau181 (a), T−tau (b), and NfL (c), and between P−tau181 (d), T−tau (e), and NfL. *P* values were derived from linear regression models adjusted for *APOE* genotype, age, sex, education, BMI, smoking status, hypertension, diabetes, and dyslipidemia. Yellow, blue, and purple points represent *APOE2*, *APOE3*, and *APOE4* carriers. Panels (f-g) display the interactions between Aβ42 and NfL: (f) with P−tau181 and (g) with T−tau. Solid and dashed lines represent NfL below and above the median, respectively, with point color indicating NfL levels. *P* values for the interaction between Aβ42 and NfL were derived from linear regression models adjusted for *APOE* genotype, age, sex, years of education, BMI, smoking status, hypertension, diabetes, and dyslipidemia.
**Supplementary figure 3: Differentially abundant proteins by amyloid status across *APOE* genotypes** (a) Scatter plot showing the relationship between Aβ42 and P-tau181, with point color indicating the P-tau181/Aβ42 ratio. (b-d) Volcano plots showing differentially abundant proteins (*P* < 0.01) by amyloid status in *APOE2* carriers (b), *APOE3* individuals (c), and *APOE4* carriers (d). *P* values were derived from linear regression models adjusted for age, sex, years of education, BMI, smoking status, hypertension, diabetes, and dyslipidemia.

Supplementary Files

This is a list of supplementary files associated with this preprint. Click to download.

- Ning2026SICSFproteomicsignaturesrevealAPOEgenotypedependentlipidandimmuneprofilesincognitivelyunimpairedelderly.xlsx

## Figures and Tables

**Table 1.** Demographics of CSF samples stratified by *APOE* genotype in the MCSA cohort

	*APOE2* (N=49)	*APOE3* (N=48)	*APOE4* (N=48)	All subjects (N=145)	*P* value
Age (years)	76.13 (6.51) *	76.96 (6.05)	76.86 (6.27)	76.64 (6.25)	0.72
Sex (Male)	31 (63.3%)	30 (62.5%)	30 (62.5%)	91 (62.8%)	1.00
Years of education	14.12 (3.13)	14.56 (2.25)	14.56 (2.53)	14.41 (2.66)	0.45
BMI (Kg/m^2^)	30.05 (5.55)	27.76 (3.59)	26.92 (4.62)	28.25 (4.82)	0.018
Smoking (former or current)	23 (46.9%)	29 (60.4%)	25 (52.1%)	77 (53.1%)	0.40
Hypertension	38 (77.6%)	37 (77.1%)	32 (66.7%)	107 (73.8%)	0.42
Diabetes	9 (18.4%)	9 (18.8%)	10 (20.8%)	28 (19.3%)	0.97
Dyslipidemia	32 (65.3%)	41 (85.4%)	42 (87.5%)	115 (79.3%)	0.015

* Mean (SD) or no. (%) of subjects

**Table 2.** Demographics of CSF samples stratified by *APOE* genotype in the replication cohorts

ADNI	*APOE2* (N=22)	*APOE3* (N=102)	*APOE4* (N=40)	All subjects (N=164)	*P* value
Age (years)	73.35 (5.12) *	74.79 (6.03)	74.11 (5.58)	86 (52.4%)	0.725
Sex (Male)	12 (54.5%)	51 (50.0%)	23 (57.5%)	74.43 (5.80)	0.459
Years of education	15.59 (3.29)	16.51 (2.52)	16.25 (2.69)	16.32 (2.67)	0.559
Emory	*APOE2* (N=20)	*APOE3* (N=59)	*APOE4* (N=25)	All subjects (N=104)	*P* value
Age (years)	65.55 (5.31) *	63.90 (7.52)	65.72 (9.04)	64.65 (7.54)	0.557
Sex (Male)	6 (30.0%)	16 (27.1%)	6 (24.0%)	28 (26.9%)	0.912
MoCA	27.45 (1.19)	27.64 (1.35)	28.28 (1.28)	27.76 (1.33)	0.061

Amsterdam	*APOE2* (N=22)	*APOE3* (N=147)	*APOE4* (N=106)	All subjects (N=275)
Age (years)	64.4 (13.1) *	66.8 (12.0)	63.9 (8.78)	65.45 (11.0)
Sex (Male)	14 (63.6%)	78 (53.1%)	56 (52.8%)	74.43 (5.80)
Years of education (median [Q1-Q3])	13 [10–13]	13 [10–14]	13 [10–14]	13 [10–14]

* Mean (SD) or no. (%) of subjects

## Data Availability

All data will be made available upon reasonable request following publication.
