# Supplementary figures and images for: Cerebrospinal fluid proteomic signatures reveal APOE genotype-dependent lipid and immune profiles in cognitively unimpaired elderly
Source: Res Sq. 2026 Feb 5:rs.3.rs-8605807. Preprint. [Version 1] doi: 10.21203/rs.3.rs-8605807/v1 (PMC12889836; doi:10.21203/rs.3.rs-8605807/v1)

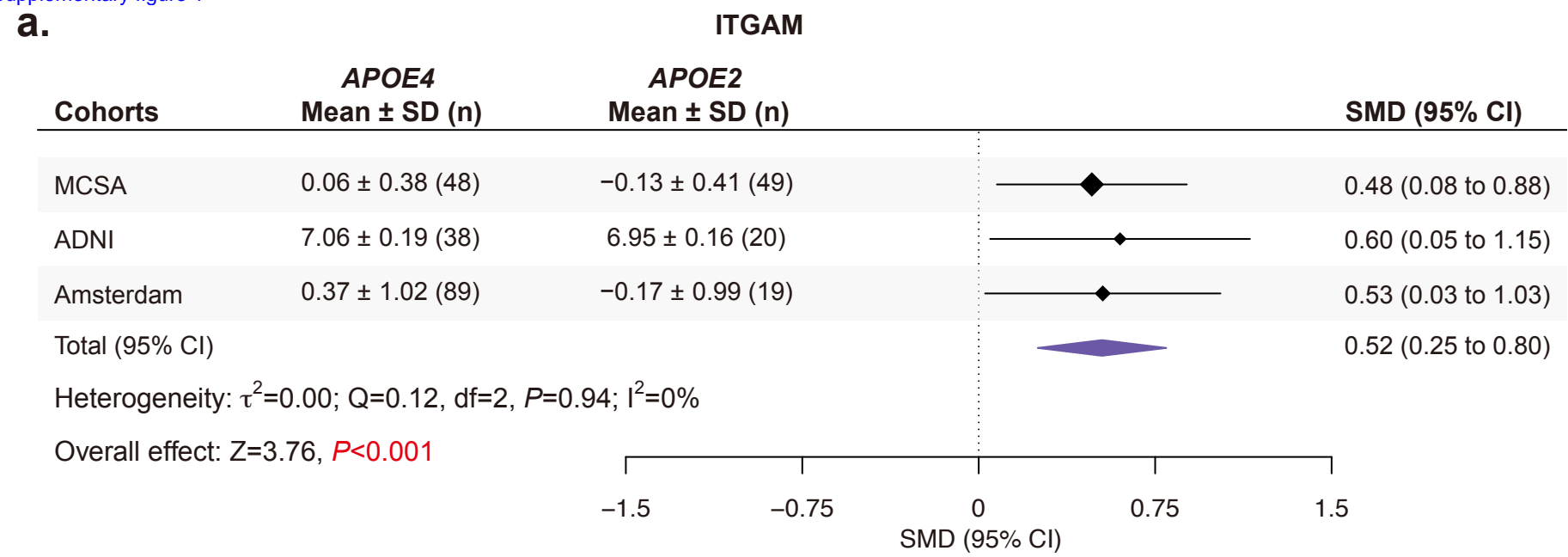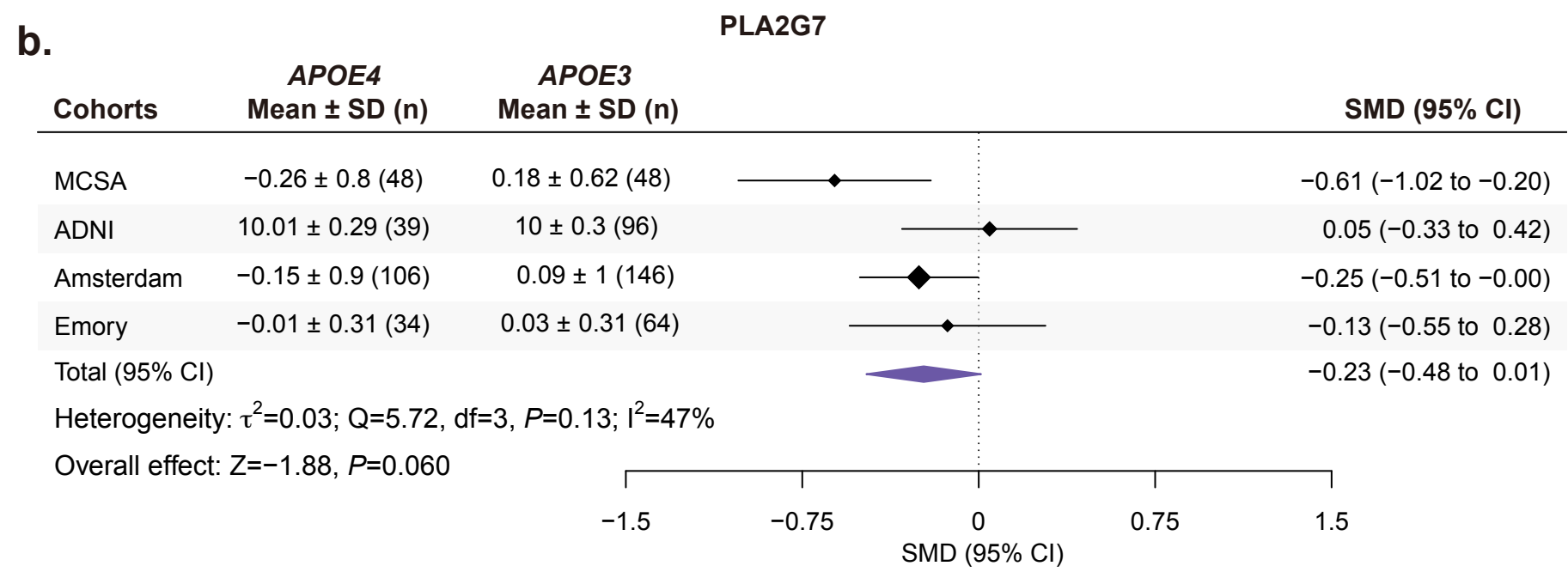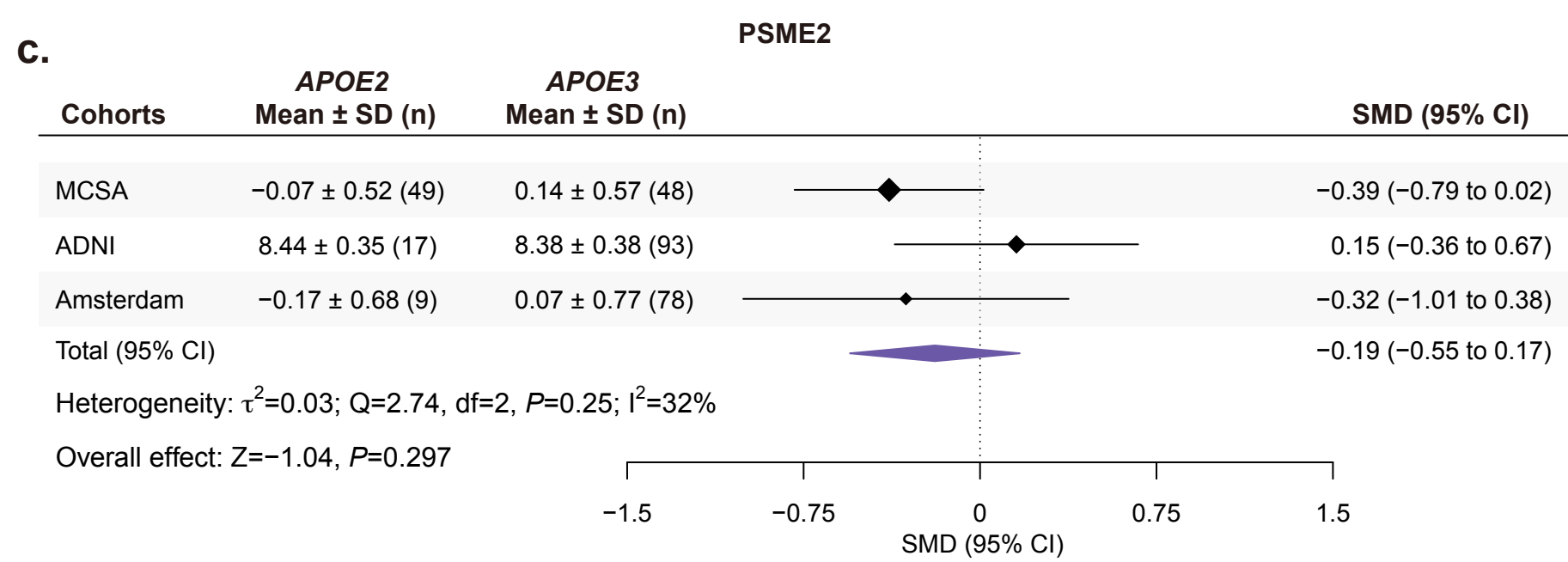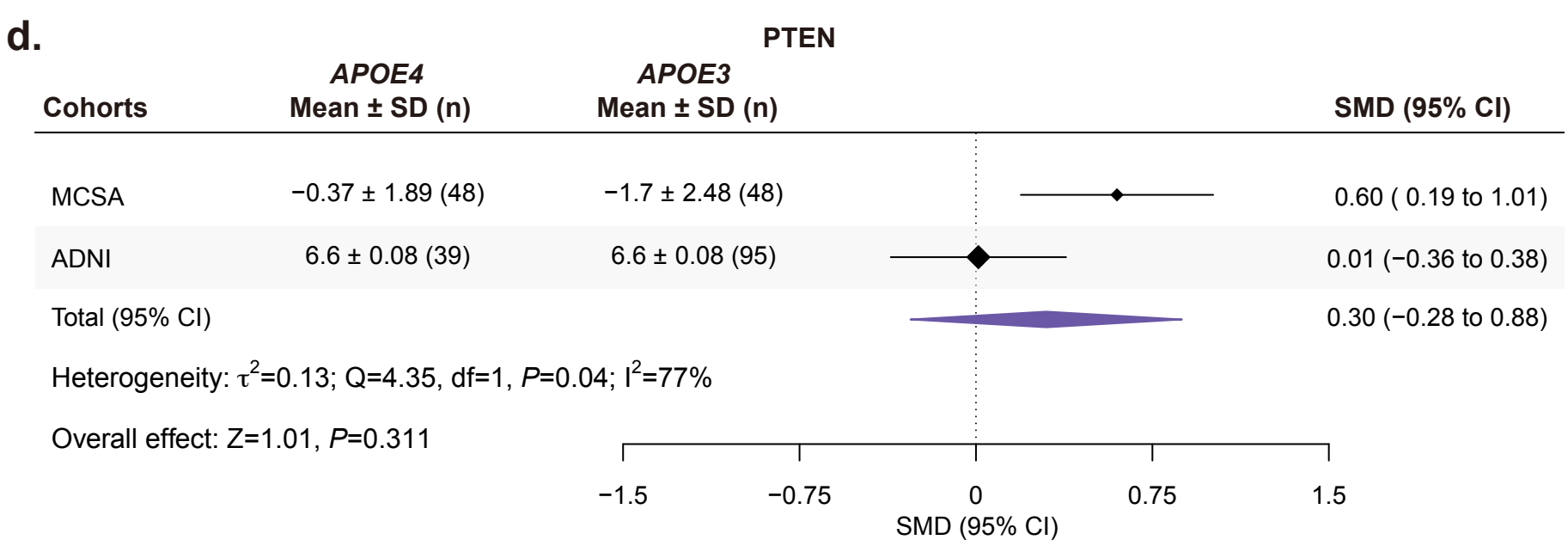

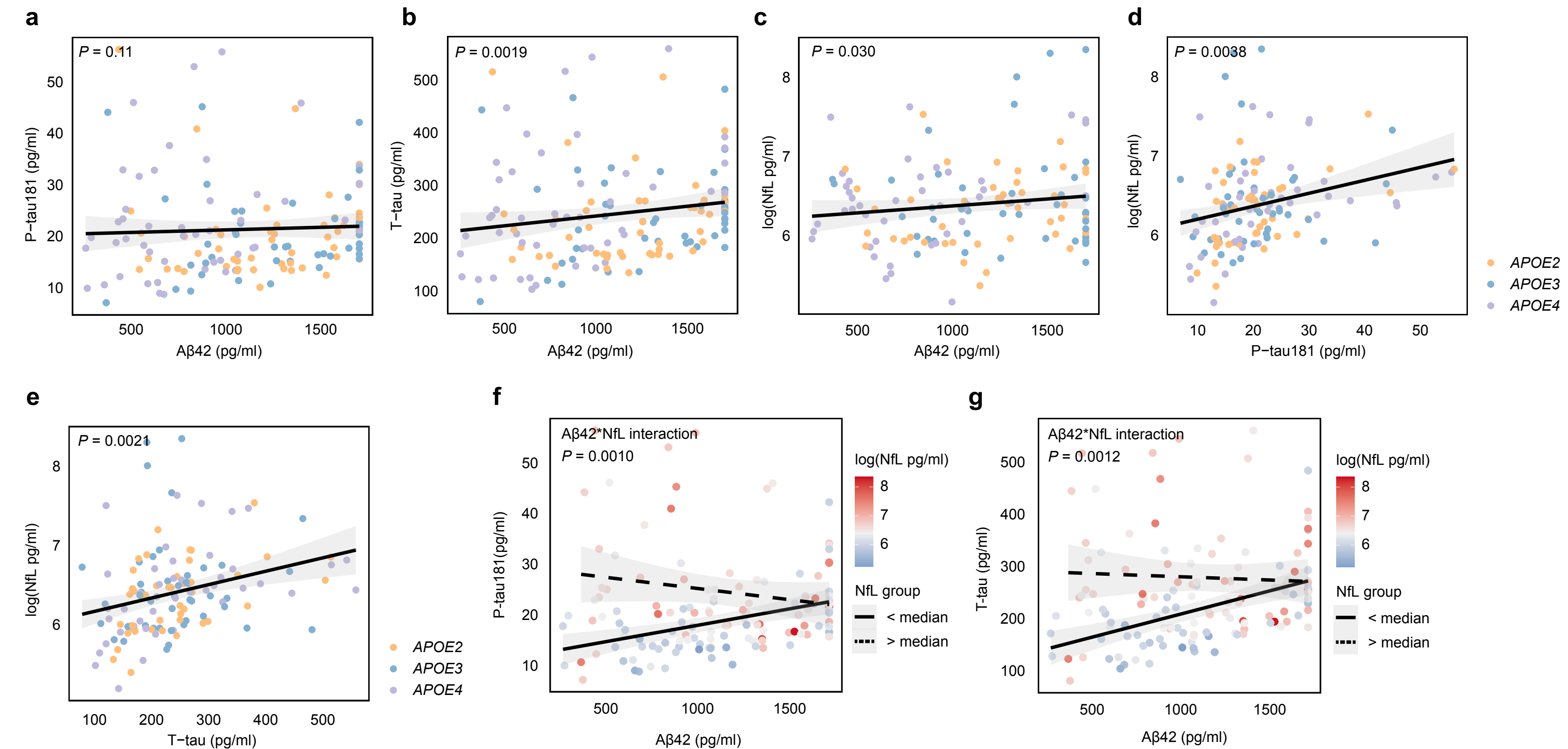

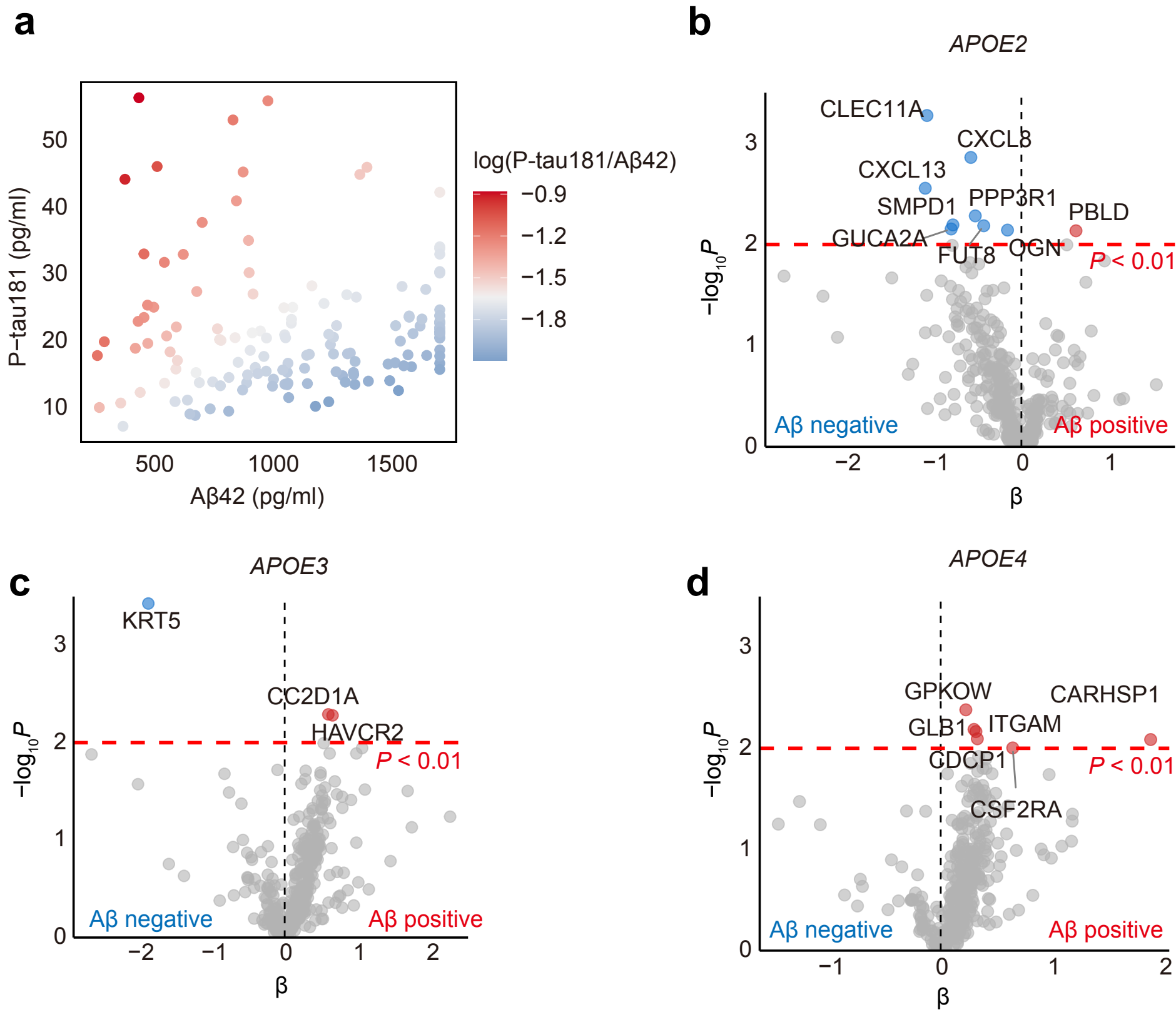

Supplement: 1 — Supplementary figure 1: Cross-cohort and cross-platform meta-analysis. Forest plot showing the standardized mean difference (SMD) of ITGAM (a), PLA2G7 (b), PSME2 (c), and PTEN (d) across different cohorts (MCSA, ADNI, Amsterdam, and Emory). SMD values represent the effect sizes for each protein, and heterogeneity was assessed across the cohorts. P values were calculated using a mixed-effects model. Supplementary figure 2: Associations among ATN biomarkers (a-e). Scatter plots show the relationships between Aβ42 and P−tau181 (a), T−tau (b), and NfL (c), and between P−tau181 (d), T−tau (e), and NfL. P values were derived from linear regression models adjusted for APOE genotype, age, sex, education, BMI, smoking status, hypertension, diabetes, and dyslipidemia. Yellow, blue, and purple points represent APOE2, APOE3, and APOE4 carriers. Panels (f-g) display the interactions between Aβ42 and NfL: (f) with P−tau181 and (g) with T−tau. Solid and dashed lines represent NfL below and above the median, respectively, with point color indicating NfL levels. P values for the interaction between Aβ42 and NfL were derived from linear regression models adjusted for APOE genotype, age, sex, years of education, BMI, smoking status, hypertension, diabetes, and dyslipidemia. Supplementary figure 3: Differentially abundant proteins by amyloid status across APOE genotypes (a) Scatter plot showing the relationship between Aβ42 and P-tau181, with point color indicating the P-tau181/Aβ42 ratio. (b-d) Volcano plots showing differentially abundant proteins (P < 0.01) by amyloid status in APOE2 carriers (b), APOE3 individuals (c), and APOE4 carriers (d). P values were derived from linear regression models adjusted for age, sex, years of education, BMI, smoking status, hypertension, diabetes, and dyslipidemia. [file NIHPPRS8605807V1-supplement-1.pdf]
